# Editorial: Frontiers in the study of ancient plant remains

**DOI:** 10.3389/fpls.2023.1177435

**Published:** 2023-03-29

**Authors:** Jianping Zhang, Ying Guan, Xinyi Liu

**Affiliations:** ^1^ Key Laboratory of Cenozoic Geology and Environment, Institute of Geology and Geophysics, Chinese Academy of Sciences, Beijing, China; ^2^ Innovation Academy for Earth Science, Chinese Academy of Sciences, Beijing, China; ^3^ Key Laboratory of Vertebrate Evolution and Human Origins of Chinese Academy of Sciences, Institute of Vertebrate Paleontology and Paleoanthropology, Chinese Academy of Sciences, Beijing, China; ^4^ Department of Anthropology, Washington University in St. Louis, St. Louis, MO, United States

**Keywords:** macrofossil and microfossil, archaeology and paleoenvironment, carbonized seeds, phytolith, pollen, starch

Scholarly curiosity about ancient plants and their roles in projected archaeology can be traced back to the 19^th^ century when archaeologists frequently encountered charred and desiccated remains at prehistoric sites in Europe and Egypt (Renfrew, 1973). Among the early explorers, Oswald von Heer’s (1865) work at Swiss Lake dwellings resonates much with modern-day inquiries and is often associated with the birthplace of paleoethnobotany. From the 1960s onwards, the study of ancient plant remains has been intricately entwined with the emerging concerns of food production and subsistence, and generally foodways in its multiple facets, dietary and symbolic, ecologic and economic, environmental and political in a range of environments can be categorized as the ‘West’, including southwestern Asia, Europe and North America (Higgs, 1972; Renfrew, 1973; Watson, 1976).

Much of the recent developments are from the global south, including East Asia, about which little was known archaeobotanically a few decades ago. The recent flourish over the last decade or so has been a transformative time in which considerable momentum was forged towards a better understanding of Asian prehistory. The consequent knowledge generated has profound implications for the understanding of the human past on a more global scale (McRostie, 2013; Liu et al., 2019; Pavlik et al., 2021; He et al., 2022). The 26 manuscripts included in this Research Topic celebrate the recent florescence and its deep roots. Collectively, the authors played a role in bringing novel lab and field-based practices into the enterprise and in bridging conceptual gaps between different theoretical strains. The interdisciplinary studies presented in the topic elucidate the spatial and temporal scales of recent development emphasizing not only on the plant itself but the humanity underlying its production and consumption.

The 26 papers in this Research Topic can be summarized into four main themes: methodological improvement, research on the relationship between ancient human activities and the environment, early food production, and paleoenvironment, paleoecology and climate changes. The methodological studies include ethnological methods, microbiological analysis, fossil morphology and stable isotope studies, etc. These advances in methodology provide a deeper understanding of the relationship between human activities and the environment.

An et al. conducted innovative ethnological research on the utilization of acorns (*Quercus* sp.) in the prehistoric period, through a case study of the production of “mook” using acorns in South Korea. The study highlights the laborious nature of acorn processing and the different methods used, due to different species and culinary traditions. This research provides new insights into the interpretation of acorn remains from prehistoric sites and is significant for the understanding of human subsistence strategy in the pre-agricultural period and the origin of agriculture (Fuller and Qin, 2010).

Liu et al. conducted microbotanical analysis on soil from anthropogenic sediments in activity areas at Tel Tsaf in the Jordan Valley, Israel. The researchers found fiber microremains, including bast fibers and the earliest evidence of cotton in the Near East (ca. 5,200-4,700 cal BC), some of which were dyed in various colors. The cotton remains probably derived from wild species originating in South Asia predating the oldest known cotton domestication in the Indus Valley by about two millennia.

Ancient plant remains, including macrofossils and microfossils, are the most important evidence for revealing issues such as human plant utilization and natural vegetation succession in prehistoric and historical periods (Piperno et al., 2004; Piperno, 2006; Zhao, 2010; Ball et al., 2016). One of the directions that the archaeobotanical (or paleoethnobotanical) community has always been working on is the improvement of caretia for fossil identification and the precisions of such caretia (Taylor et al., 2009).

Carbonized cereal grains are one of the most commonly preserved plant fossils in archaeological contexts, and the influence of the carbonization process on seed morphology is particularly worthy of attention, which is related to the accuracy of fossil identification (Yang et al., 2011; Castillo, 2018). In their article, Liu et al. highlighted the importance of the charring effect on bread wheat (*Triticum aestivum*), foxtail millet (*Setaria italica*), broomcorn millet (*Panicum miliaceum*), rice (*Oryza sativa*), and soybean (*Glycine max*) in archaeological interpretation. They found that temperature and exposure time directly affect grain size, with the grains of most species shrinking at lower temperatures and expanding rapidly at higher temperatures.

In addition to the charring effect on plant fossil morphology, Dong et al. investigated the charring effect as well as growing conditions on plant isotope compostitions. They found that the stable nitrogen isotope values of foxtail millet and broomcorn millet can shift up to 1-2 ‰ when charred, while the stable carbon isotope values change less than 0.3 ‰. They further evaluated the feasibility of using stable carbon and nitrogen isotope analysis of charred archaeobotanical remains and suggested that the stable nitrogen isotope values of charred millet seeds could provide insight into past field management practices. Both carbon and nitrogen isotope values can be informative on ancient diets, combining with other lines of evidence such as zooarcaheological and archaeobotanical infomration.

In the studies of microfossils, Yu et al. demonstrated the potential of seed starch grains as proxies for reconstructing ancient plant use. The study analyzed nuts from 40 species of four genera of Fagaceae from South China for statistical measurement and comparative analysis of starch grains. The results showed that 34 species had high accumulation of starch grains, and the morphological characteristics varied between species but were similar between species in the same infragenious section. This expansion of modern starch research and comparison helps to improve the accuracy of identifying ancient starch and deepen our understanding of ancient human plant utilization.

In the studies of phytolith morphology, Ge et al. investigated the production and morphologies of phytoliths in modern plants on the Tibetan Plateau. The study found that the major phytolith producers are Poaceae and Cyperaceae, and the production of phytolith in most samples is higher than 0.4 million grains/g. Phytolith morphotypes may indicate different hydrological conditions on the Tibetan Plateau, providing new information that will aid future phytolith analysis in the region. Furthermore, Wang et al. analyzed morphological characteristics of phytoliths from a total of 111 species from 50 families, including 73 species from 33 tree/shrub families, 31 species from 12 herb families and 7 species from 5 fern families, in the low latitudes of Southwest China. The results suggest that those phytoliths are mainly deposited *in situ* and have unique characteristics that are representative of the low-latitude subtropical monsoon climate.

Another theme that emerges from this topic is the role of subsistence strategies and environment in shaping human-plant interactions, based on the evidence from ancient plant remains. Four papers revealed that changes of human subsistence since the Neolithic were likely triggered by variations in human settlement intensity (Yang et al.), environment and climate changes of different time scales in different regions (Liu et al., Gao et al. and Jia et al.), and a review of the relationship between environmental changes and subsistence strategy from the Shunshanji cultural region in the Huai River in Central China revealed the complex interplay between human behavior and environmental changes (Qiu and Rao).

Human impacts on physical environment has been well discussed in recent research. Such relationship can be sometimes altered. Dai et al. illustrated how the subsistence of the Neolithic people in South Hangzhou Bay, China, changed as the landscape evolved from a largely marine-influenced setting to a coastal plain. Initially, the semi-enclosed landscape provided resources for hunting, gathering, and possibly some incipient rice cultivation. As the Yaojiang Valley wetland desalinized, rice farming became more common and increasingly important in the Neolithic people’s diet, suggesting that the shift of subsistence from hunting-gathering to rice farming was an adaptive strategy to the changing landscape. Zhang et al. also presented a case study of the interaction between early human activities and landscape evolution in the piedmont of Taihang Mountain in China. The study found that shrinking of flooded areas due to river downcutting and watercourse fixation from the late Longshan culture (after 4000 BP) created a suitable habitat for human settlement, leading to large-scale human migration to the area and the growth of early civilization.

The papers in this tropic also offer new insights into the early development of agriculture and food production from the late Pleistocene to the historical epoch in East Asia. Wu et al. examined the starch grains found in dental calculus from Fuyan Cave hominins in Daoxian, South China, believed to be the earliest modern humans in East Asia. The results showed that early modern humans in East Asia consumed a diet consisting of acorns, roots, tubers, grass seeds, and other plant resources during Marine Isotope Stage 5 (MIS5) between 120 and 80 ka. The findings suggested that acorns may have played an important role in subsistence strategies and may have been a part of the long-lasting tradition of using these plants during the late Pleistocene in China.

With the arrival of the late Neolithic, millets (*Setaria italica* and *Panicum miliaceum*) paired with wheat (*Triticum aestivum*), barley (*Hordeum vulgare*), buckwheat (*Fagopyrum esculentum*) and beans (Leguminosae) became staple crops in human subsistence that lasting to the historic period of northern China (Yang et al., He et al., Liao et al., Lu et al., Li et al.). While in southern China, rice remains were widespread and were often paired with millets, job’s tears (*Coix lacryma-jobi*), lotus roots (*Nelumbo nucifera*), possibly Chinese yam tubers (*Dioscorea panthainca*), acorns (*Quercus* sp.), and beans (*Vigna* sp. or/and *Vicia* sp.) (Khan et al.) and other northern dryland crops, such as wheat and barley (Yang et al.). These newly introduced crops, along with rice, formed a multi-cropping system that served as the part of the economic foundation for the rise of the regional culture, such as Chu kingdom during Eastern Zhou Dynasty (770-256 BCE). The two distinct agricultural systems in northern and southern China, which were complementary in terms of diet and had positive interactions and feedback, led to the sustainable intensification of agriculture and the emergence of complex societies and early states in the Yellow and Yangtze Rivers (He et al., 2022).

In Southeast Asia, Deng et al. provided evidence that Neolithic farmers in Taiwan cultivated both rice and foxtail millet at least 4,500 years ago. These findings support the hypothesis that proto-Austronesian people brought their crops and cultural traditions from the southeast coast of mainland China to Taiwan 4,800 years ago and beyond into Island Southeast Asia. Wang et al. further analyzed micro-plant remains from three Neolithic sites in Ha Long Bay, Vietnam and found evidence of the earliest co-cropping of rice and foxtail millet in the region 4000 years BP. This study also revealed the diversity of subsistence strategies among different cultural groups in Vietnam.

One of the research areas in plant remains that has seen major advances in the recent decades is paleoclimate and paleoenvironment. In this theme, Li et al. found that the different subtropical biomes could be distinguished by phytolith assemblages, with tree coverage being best represented by topsoil phytoliths, and grass silica short cell phytoliths (GSSCP) occurring more frequently in open habitats at higher elevations. It was also suggested that human-induced deforestation could increase the frequency of GSSCP.

Based on the modelling approach (The Regional Estimates of Vegetation Abundance from Large Sites, REVEALS), Niu et al. reconstructed the past regional plant cover and explored the influence of climate, fire, and human activity on vegetation dynamics. The results show that long-term vegetation dynamics were primarily driven by the East Asia Summer Monsoon and precipitation variations, but were also impacted by fire frequency and human activity. This study concluded that the East Asian Summer Monsoon and precipitation were the main drivers of vegetation change, while fire had a greater impact than human activity.

Overall, the 26 papers included in this topic demonstrate the rich diversity of human-plant interactions throughout history and the importance of studying ancient plant remains to better understand human culture and subsistence practices, resonating with the recent momentum gained in the field of Archaeology and Paleoenvironment (Cappers and Neef, 2021). This collection of papers highlights the frontiers in the study of ancient plant remains and sets the stage for future research in this exciting and dynamic field.

As introduced, one of the consequences of the recent development of paleoethnobotany in Asia has been to encourage pluralism in understanding human-environment interactions and biodiversity. These recent progress serves to undermine some of the assumptions we have held for long based merely on western conditions, including some of the basic concepts such as agriculture and pastoralism. In this context, research focus on past challenges has a 21^st^-century resonance and applicability. There has been an impressive development in understanding the underlying biology of plant morphology and their responses to human activities, as showcased by this Research Topic. Nonetheless, their applications in social and cultural frameworks have yet to be fully explored. Excellent work in this domain is nevertheless limited by the lack of a general theoretical structure to recognize how plant enters the moral and social intentions of individuals and communities. This is indeed a new frontier in which future research will bolster our current strengths while bridging the gap between the biology of ancient plants and how they were used to maintain social relations.

## Author contributions

JZ wrote the first draft of the editorial. YG and XL commented and revised the draft. All authors agreed with the final draft.
